# Immunoglobulin E (IgE)-Mediated Multiple Myeloma Presenting as Pleural Effusion

**DOI:** 10.7759/cureus.45802

**Published:** 2023-09-22

**Authors:** Sarah Nisar, Anees Ur Rehman, Adil Khan, Noor Wahab, Syed Safiullah Shah

**Affiliations:** 1 Internal Medicine, Khyber Medical College, Peshawar, PAK; 2 Medicine, Ayub Medical College, Peshawar, PAK; 3 Medicine, Combined Military Hospital, Abbotabad, PAK; 4 Internal Medicine, Ayub Teaching Hospital, Abbottabad, PAK

**Keywords:** diagnosis of multiple myeloma, immunoglobulin e, immunoglobulin e-mediated multiple myeloma, hypercalcemia, bone marrow biopsy, lytic lesions, pleural effusion, multiple myeloma

## Abstract

Multiple myeloma (MM) is a disease characterized by the abnormal production of plasma cells. These plasma cells have the property to produce immunoglobulins (Ig), on the basis of which MM can be classified. Immunoglobulin G is responsible for most of the cases, while IgE mediates the rarest form of MM. Since the first case was reported in 1967, knowledge regarding IgE-mediated MM is based on individual case reports. Based on the information available, it is thought that IgE-mediated MM presents clinically with the involvement of renal, bone, and hematological symptoms, which are the same as any other myeloma. However, the MM rarely involves the pleura, leading to malignant pleural effusion. We present a case of IgE-mediated MM with a unique feature of pleural effusion. The patient presented with constitutional symptoms of MM, which were followed by lab investigations revealing low hemoglobin, hypercalcemia, and high creatinine levels. An incidental computed tomography angiography (CTA) revealed lytic lesions in the spine. This was followed by skull and chest X-rays as part of the workup to determine the extent of the disease. It revealed further lytic lesions in the skull, humerus, and scapula, along with pleural effusion. This led to the suspicion of MM, which was ultimately confirmed by serum protein electrophoresis and a bone marrow biopsy. The patient was started on a triple regimen of bortezomib, thalidomide, and dexamethasone, which led to substantial improvement in his symptoms.

## Introduction

Overall, hematologic malignancies account for 6.5% of the cancer burden worldwide [1]. Among these, multiple myeloma (MM) accounts for 10% of the cases [2].

Multiple myeloma is a plasma cell malignancy responsible for aberrant immunoglobulin (Ig) protein production. Ultimately, these proteins deposit in multiple areas of the body, giving the disease the potential to present with a wide range of signs and symptoms [3]. Multiple myeloma can lead to any monoclonal gammopathy involving IgG (52%), IgA (21%), IgD (2%), and rarely IgE (0.1%) antibodies [4]. The first case of IgE-mediated MM was reported in 1967. Since then, only 63 cases have been added to the literature [4]. As with any other myeloma, IgE-mediated MM can present with symptoms of renal insufficiency and hematological and bone complications [5]. However, myelomas leading to pleural infiltrations are a rare occurrence (1%) [6]. We present a case in which a patient with IgE-mediated MM presented with signs and symptoms of pleural effusion.

## Case presentation

A 55-year-old male presented at our hospital with a collection of concerning symptoms, including an unexplained weight loss of 15 kg, epigastric discomfort, dyspnea, diminished appetite, and palpitations. His medical history revealed ischemic heart disease with non-obstructive coronary artery disease and myocardial bridging as observed in angiography.

Upon admission, the patient displayed symptoms of dyspnea, tachycardia, and tenderness in the epigastric region. Laboratory assessments unveiled anemia, hypercalcemia, and elevated serum creatinine levels, as outlined in Table 1.

**Table 1:** The patient's baseline CBC, CRP, and RFT HCT: hematocrit; MCV: mean corpuscular volume; CRP: C-reactive protein; RFT: renal function test; WBC: white blood cell; CBC: complete blood count; HCT: hematocrit; RBC: red blood cell

Parameter	Values: Day 1	Values: Day 2	Values: Day 3	Reference Range
WBC	7.1	5.2	11.7	4.5-11x10^9/L
Hemoglobin	7.4	9.1	6.6	14-16.5 g/dl
RBC	3.38	3.72	2.93	4.45x10^12/L
HCT	23.4	27.2	20.3	36-54%
MCV	71.4	73.1	69.5	76-96fL
Platelets	335	203	271	150-400x10^9/L
CRP	16.31	17.1	17	Less than 5.0 mg/L
Calcium	12.78	11.2	9.8	8.0´-10 mg/dL
Blood urea	189.8	194.6	188	10-50 mg/dL
Osmolarity	291	287	285	280-295 mmol/L
Creatinine	4.6	3.1	2.8	0.64-1.2 mg/dL
Creatinine clearance	60	77	90	97-137 ml/min
Sodium	141	130.0	137.1	135-150 mmol/L
Potassium	3.5	4.1	4.9	3.5-5.1 mmol/L
Chloride	110	99.7	109	96-112 mmol/L

Delving deeper into the anemia, elevated serum ferritin levels were discovered, while folate and B12 levels remained within the normal range (Tables 2, 3).

**Table 2:** The patient's calcium profile PTH: parathyroid hormone; PTHrP: parathyroid hormone-related protein

Parameter	Values	Reference Range
Calcium	12.78	8-10mg/dl
PTH	4.5	Less than 5pg/ml
PTHrp	1	Less than 1.1 pmol/l

**Table 3:** The patient's anemic profile LDH: lactate dehydrogenase

Parameter	Values	Reference Range
Total iron concentration	43	50-17 pg/µl
Total iron binding capacity	214	286-569 µg/dl
Ferritin	408	11-307 pg/ml
Iron saturation	9	11-42 %
Transferrin	180	192-382 pg/ml
B12	345	213-816 pg/ml
Folic acid	8	7-31.4 pg/ml
LDH	176	140-280 U/l
Total bilirubin	1.1	0.3 to 1.0 mg/dL
Direct (conjugated) bilirubin	0.25	0.0 to 0.3 mg/dL
Indirect (unconjugated) bilirubin	0.77	0.2 to 0.8 mg/dL

Positive occult blood results from stool testing prompted subsequent upper and lower gastrointestinal (GI) colonoscopies to investigate potential sources of bleeding. Both procedures yielded predominantly normal results.

**Figure 1:**
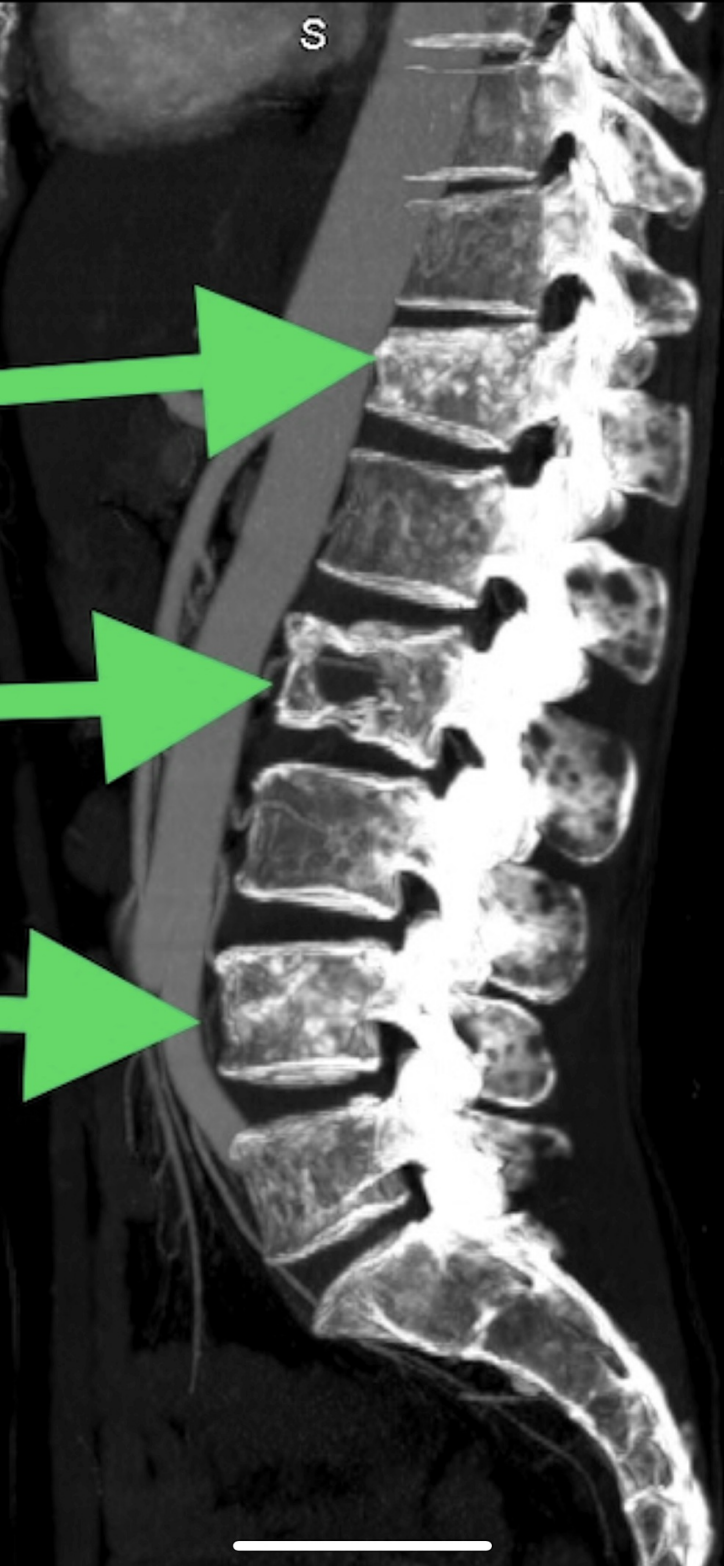
A computed tomography angiography of the abdomen (sagittal plane); the arrows indicate several lytic lesions in the spine

**Figure 2:**
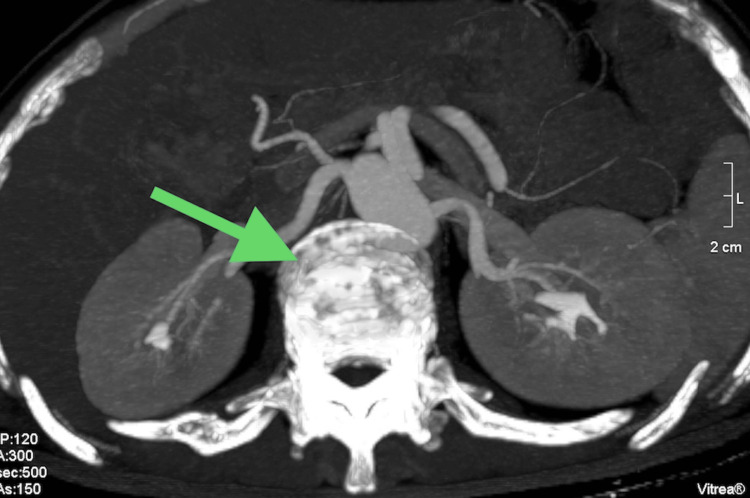
A computed tomography angiography of the abdomen (transverse plane); the arrow indicates a lytic lesion in the spine

**Figure 3:**
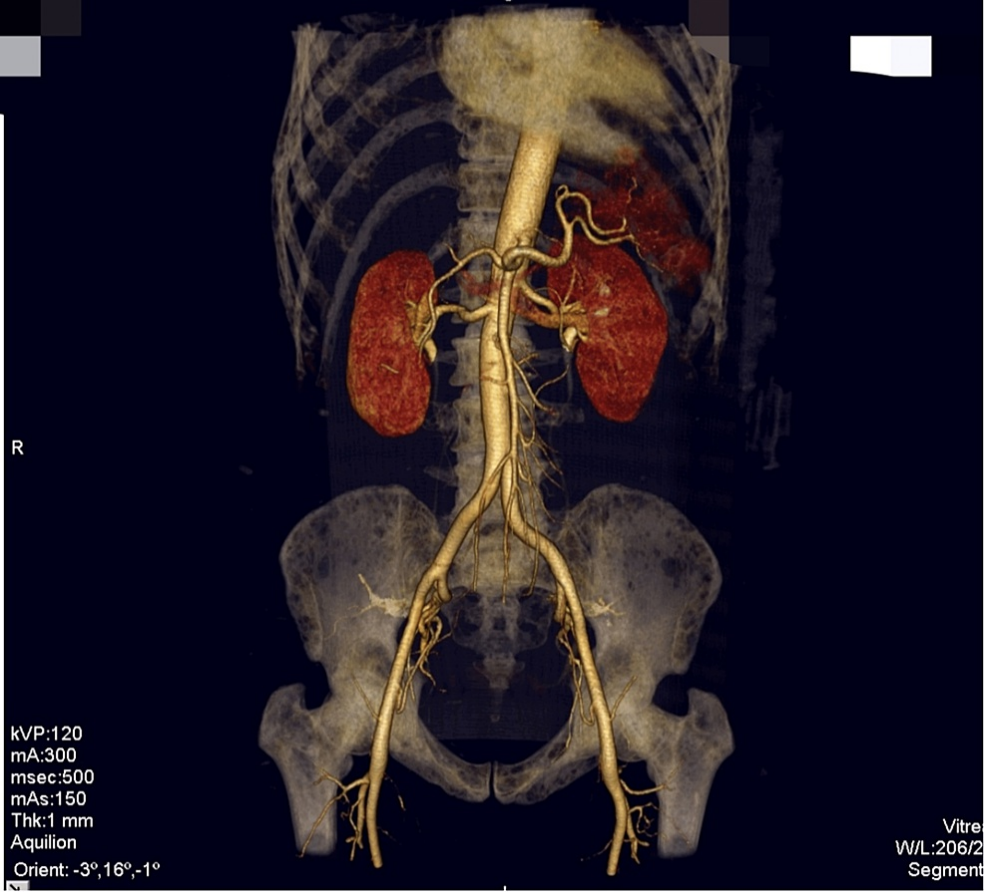
Computed tomography angiography of the abdomen indicating lytic lesions in the bone.

The emergence of postprandial abdominal pain prompted consideration of mesenteric ischemia, leading to a computed tomography angiography (CTA) scan. While this scan did not reveal mesenteric abnormalities, it surprisingly uncovered extensive lytic lesions in the spine (Figures 1-3).

**Figure 4:**
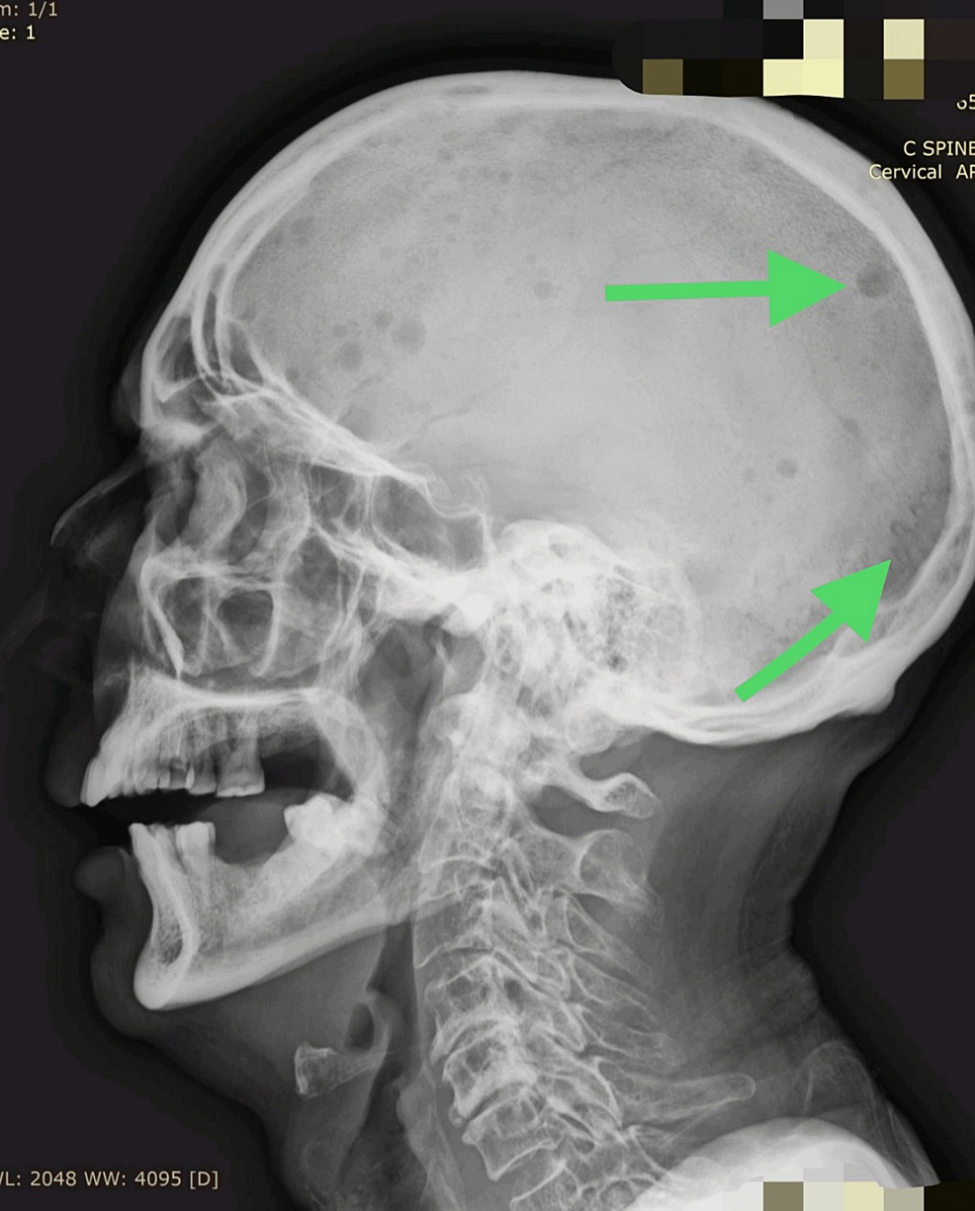
An X-ray of the skull (lateral view) with the arrows indicating lytic lesions in the skull

Expanding upon the findings of hypercalcemia in the lab results and the presence of lytic lesions in imaging, an X-ray of the skull (lateral view) revealed further lytic lesions (Figure 4).

**Figure 5:**
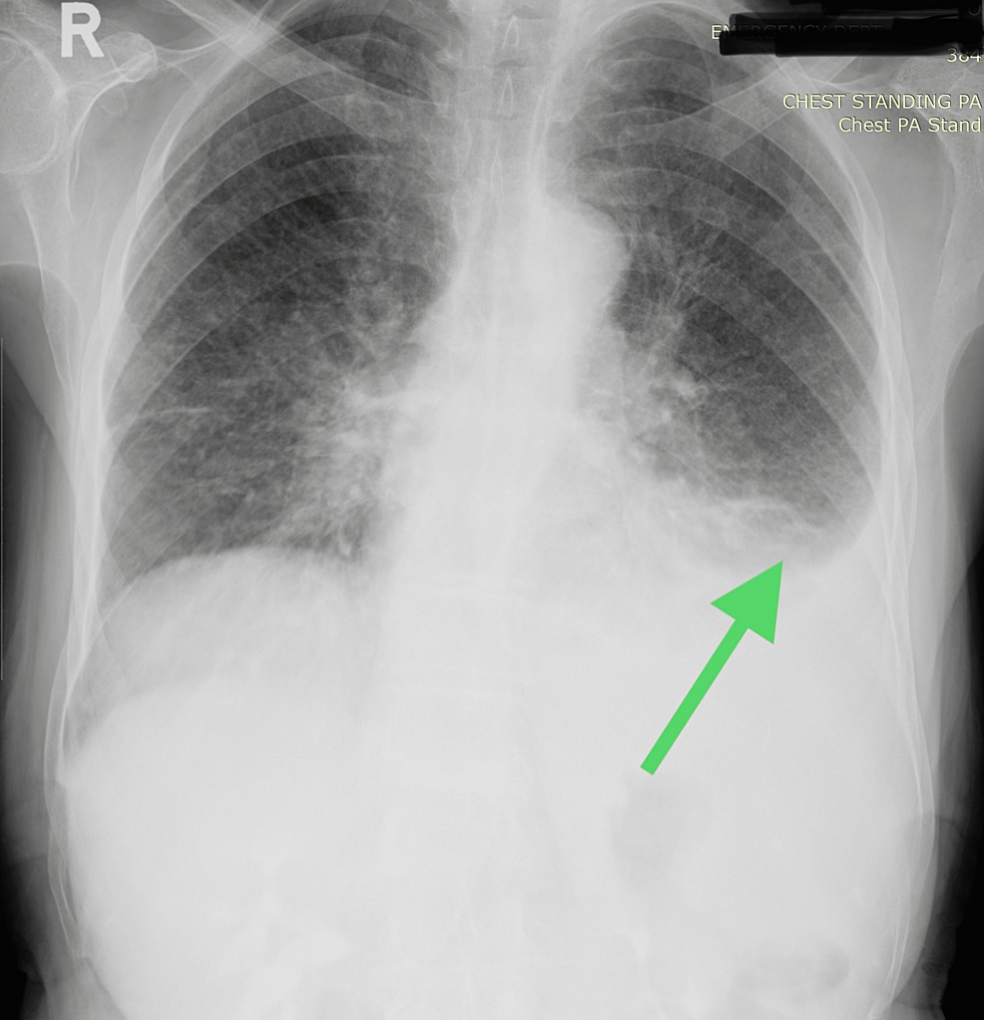
Chest X-ray; the arrow indicates left-sided pleural effusion

**Figure 6:**
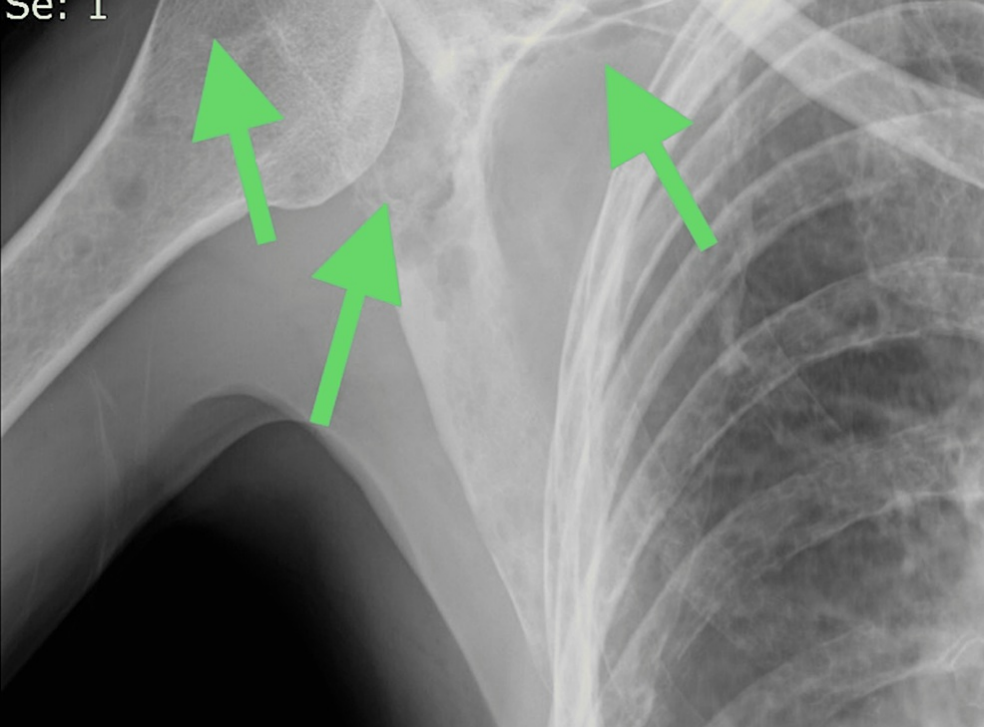
Chest and shoulder X-ray; the arrows indicate lytic lesions in the humerus and scapula.

Subsequent chest X-rays revealed pleural effusion, leading to a pleural tap that confirmed the presence of exudative fluid. A follow-up CT scan a month later revealed the reaccumulation of pleural fluid. Notably, the chest X-ray also showed lytic lesions in the humerus and scapula (Figures 5, 6).

**Figure 7:**
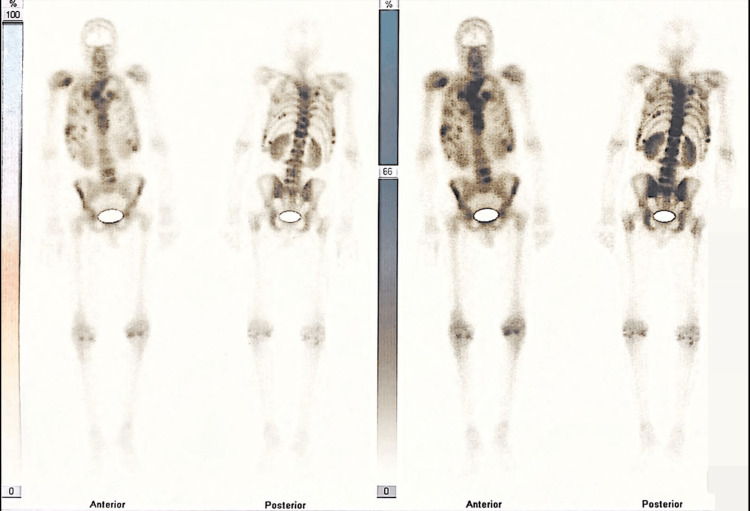
A bone scan shows decreased radiotracer uptake.

Given the recurrent hypercalcemia, lytic lesions, abnormal creatinine levels, and anemia, a bone scan was conducted, revealing significant lytic lesions in the spine. Reduced radiotracer uptake, characteristic of a cold bone scan, raised suspicions of multiple myeloma (Figure 7).

This prompted serum protein electrophoresis (SPEP), which indicated a substantial homogenous paraprotein band (6.3g), further strengthening the suspicion of multiple myeloma. Serum immunofixation evidenced a monoclonal band attributable to IgE kappa with an IgE serum concentration of 3,572,95 kU/L. Hence, the diagnosis was refined to monoclonal IgE kappa multiple myeloma, which was also in accordance with the International Myeloma Working Group (IMWG) criteria [7].

To solidify the diagnosis, a bone marrow biopsy was performed, showing extensive infiltration of plasma cells with 80% involvement.

The patient, due to his significant history of abnormal cardiac and renal profiles, was deemed unfit for stem cell transplantation; however, the therapeutic intervention was commenced with a bortezomib-based triplet regimen for remission induction, specifically bortezomib (a proteasome inhibitor) given at a dosage of 1.3mg twice a week, thalidomide (an immune modulator) at 200mg once a week, and dexamethasone (a glucocorticoid) at 30mg once a week, respectively. This regimen was followed for eight cycles. Adjunctive therapy was also implemented in the form of bisphosphonates (zolendronic acid) to counter skeletal events (e.g., hypercalcemia, spinal cord compression, pathologic fracture, surgery, radiation). Anemia correction was administered through transfusions.

The patient exhibited substantial improvement under this regimen. Treatment was sustained for three months, after which the patient was advised to undergo follow-up assessments, including serial X-rays to monitor pleural effusion, a complete blood count (CBC), chemical profile 7 (with a specific focus on blood urea nitrogen (BUN), serum creatinine, calcium, and uric acid) (Table 4), and SPEP to monitor IgE levels.

**Table 4:** The patient's blood and chemical profile during follow-up assessments

Parameter	Values	Reference range
Hemoglobin	14.7	14-16.5 g/dl
Blood urea nitrogen (BUN)	20.1	6-24 mg/dl
Creatinine	1.1	0.64-1.2 mg/dL
Calcium	9.9	8-10mg/dl
Blood urea	43.7	10-50 mg/dL

Lenalidomide, 50 mg/day, was introduced as maintenance therapy. The response was measured by a bone marrow biopsy, which revealed the absence of clonal and plasma cells; additionally, serum and urine reduction of plasma cells was also a promising sign, which was in accordance with the "very good partial response" (VGPR) of the IMWG uniform response criteria [7].

## Discussion

Immunoglobulin E-mediated MM is a rare condition characterized by abnormal proliferation of plasma cells, leading to the deposition of IgE in various distinct regions of the body. This was first demonstrated by Johannesen and Bennich in 1967 [8].

As of now, only individual cases have been reported in the literature, but it has enabled us to ascertain different points related to this rare disease [8]. According to a review by Heijl et al., IgE-mediated MM has a slight predominance in men as compared to women. Similarly, the mean age of onset was found to be 67 years. The common complications of patients with IgE-mediated MM include anemia (44%), renal insufficiency (24%), and hypercalcemia (18%), due to which the patients mostly present with tiredness, shortness of breath, bone pain, stomach pain, and polyurea. This clinical presentation does not vary much in regard to other myelomas; however, the severity of these problems is much worse in cases of IgE-mediated MM [8,9]. In our case, the patient was also a middle-aged male who presented with weight loss, epigastric discomfort, dyspnea, loss of appetite, and worsening palpitations.

Rarely, cases of MM may involve the pleura, giving rise to malignant pleural effusion. This can be proved by pleural fluid analysis showing increased levels of proteins (greater than 2 g/dl) and lymphocytosis of greater than 1,000 white blood cells per cubic millimeter, respectively. Similarly, a pleural biopsy indicating thickening, nodular lesions in the parietal pleura, or segmental atelectasis also indicates malignant involvement of the pleura. As per Ghorbel et al., there have been about 80 cases of myelomatous pleural effusion reported as of 2015 [10,11]. On the other hand, according to Ana et al., the most common causes of PE in patients with MM are congestive heart failure, renal failure, parapneumonic effusion, and amyloidosis. Other causes can be hypoalbuminemia, pulmonary embolism, secondary neoplasm, and lymphatic obstruction with chylothorax. In less than 1% of cases, the effusion is a direct result of MM, hence called myelomatous pleural effusion (MPE) [12]. Eighty percent of cases of pleural effusion have been associated with IgA [12]. However, in our case, it was IgE-mediated.

Patients with MM are primarily assessed on levels of serum hemoglobin, calcium, and creatinine for anemia, bone involvement, and renal failure, respectively. A bone marrow aspirate may reveal abnormal plasma cells, and its immunohistochemical studies are essential for distinguishing the type of Ig involved. Similarly, serum free light chain levels and electrophoresis with immunofixation are vital for determining the extent of immunoglobulins involved. Full-body skeletal imaging with CT, PET, or MRI is required for evaluation of the lytic lesions spread throughout the body [3-5]. In our case, the patient underwent extensive investigations, which revealed low hemoglobin levels, deranged serum calcium, and creatinine. Similarly, an incidental CT scan revealed lytic lesions in the spine. These findings led to a suspicion of a hemolytic cause behind his symptoms. Ultimately, the diagnosis of IgE-mediated MM was confirmed by serum protein electrophoresis and a bone marrow biopsy.

Previously, the diagnosis of IgE-mediated MM was associated with a poor prognosis, and the survival limit was considered to be only about 12.5 months [4,13]. This could be attributed to the fact that this condition is extremely rare, with limited treatment options available. However, the introduction of new proteasome inhibitors, like bortezomib, to the previous treatment regimens has changed the dynamics [13,14]. Our patient was treated successfully with a regimen of bortezomib, thalidomide, and dexamethasone. This is followed by maintenance therapy, for which lenalidomide and thalidomide are the most commonly used drugs [7]. The response to therapy is usually measured by the IMWG response criteria [7]. Our patient was treated with lenalidomide as maintenance therapy.

## Conclusions

In conclusion, we report a rare case of IgE-mediated MM in which the patient presented with a unique feature of pleural effusion along with the other constitutional symptoms of MM. Knowledge regarding cases of IgE MM presenting as other myelomas can be found in the literature; however, cases reporting pleural involvement as a complication of IgE-mediated MM are rather rare. Therefore, it would be important to document cases of this entity to better establish the various ways this rare but lethal disease may present.
